# Health care impact of implementing a clinical pathway for acute care of pediatric concussion: a stepped wedge, cluster randomised trial

**DOI:** 10.1007/s43678-023-00530-1

**Published:** 2023-06-23

**Authors:** Keith Owen Yeates, Karen M. Barlow, Bruce Wright, Ken Tang, Olesya Barrett, Edward Berdusco, Amanda M. Black, Brenda Clark, Alf Conradi, Heather Godfrey, Ashley T. Kolstad, Anh Ly, Angelo Mikrogianakis, Ross Purser, Kathryn Schneider, Antonia S. Stang, Roger Zemek, Jennifer D. Zwicker, David W. Johnson

**Affiliations:** 1 Department of Psychology, University of Calgary, 2500 University Dr. NW, Calgary, AB T2N1N4 Canada; 2 Alberta Children’s Hospital Research Institute, University of Calgary, Calgary, AB Canada; 3 Hotchkiss Brain Institute, University of Calgary, Calgary, AB Canada; 4 Child Health Research Centre, Queensland Children’s Hospital, University of Queensland, South Brisbane, Australia; 5 Department of Pediatrics, University of Calgary, Calgary, AB Canada; 6 Department of Pediatrics, University of Alberta, Edmonton, AB Canada; 7 Women’s and Children’s Health Research Institute, University of Alberta, Edmonton, AB Canada; 8 Independent Statistical Consulting, Vancouver, BC Canada; 9 Alberta Health Services, Calgary, AB Canada; 10 Departments of Emergency Medicine and Family Medicine, University of Alberta, Edmonton, AB Canada; 11 O’Brien Institute for Public Health, University of Calgary, Calgary, AB Canada; 12 Faculty of Kinesiology, Sport Injury Prevention Research Centre, University of Calgary, Calgary, AB Canada; 13 Department of Emergency Medicine, Alberta Children’s Hospital, Calgary, AB Canada; 14 Department of Pediatrics, McMaster University, Hamilton, ON Canada; 15 Department of Emergency Medicine, Grey Nuns Hospital, Edmonton, AB Canada; 16 Department of Community Health Sciences, University of Calgary, Calgary, AB Canada; 17 Department of Emergency Medicine, University of Calgary, Calgary, AB Canada; 18 Departments of Pediatrics and Emergency Medicine, University of Ottawa, Ottawa, Canada; 19 Children’s Hospital of Eastern Ontario Research Institute, Ottawa, ON Canada; 20 School of Public Policy, University of Calgary, Calgary, AB Canada; 21 Faculty of Kinesiology, University of Calgary, Calgary, AB Canada

**Keywords:** Pediatric, Traumatic brain injury, Clinical pathway, Utilization, Pédiatrique, Traumatisme cérébral, Voie Clinique, Utilisation

## Abstract

**Objectives:**

To test the effects of actively implementing a clinical pathway for acute care of pediatric concussion on health care utilization and costs.

**Methods:**

Stepped wedge, cluster randomized trial of a clinical pathway, conducted in 5 emergency departments (ED) in Alberta, Canada from February 1 to November 30, 2019. The clinical pathway emphasized standardized assessment of risk for persistent symptoms, provision of consistent information to patients and families, and referral for outpatient follow-up. De-identified administrative data measured 6 outcomes: ED return visits; outpatient follow-up visits; length of ED stay, including total time, time from triage to physician initial assessment, and time from physician initial assessment to disposition; and total physician claims in an episode of care.

**Results:**

A total of 2878 unique patients (1164 female, 1713 male) aged 5–17 years (median 11.00, IQR 8, 14) met case criteria. They completed 3009 visits to the 5 sites and 781 follow-up visits to outpatient care, constituting 2910 episodes of care. Implementation did not alter the likelihood of an ED return visit (OR 0.77, 95% CI 0.39, 1.52), but increased the likelihood of outpatient follow-up visits (OR 1.84, 95% CI 1.19, 2.85). Total length of ED stay was unchanged, but time from physician initial assessment to disposition decreased significantly (mean change − 23.76 min, 95% CI − 37.99, − 9.52). Total physician claims increased significantly at only 1 of 5 sites.

**Conclusions:**

Implementation of a clinical pathway in the ED increased outpatient follow-up and reduced the time from physician initial assessment to disposition, without increasing physician costs. Implementation of a clinical pathway can align acute care of pediatric concussion more closely with existing clinical practice guidelines while making care more efficient.

**Trial registration:**

ClinicalTrials.gov NCT05095012.

**Supplementary Information:**

The online version contains supplementary material available at 10.1007/s43678-023-00530-1.

## Abstrait

**Objectifs:**

Mettre à l’essai les effets de la mise en œuvre active d’une voie clinique pour le traitement aigu des commotions cérébrales chez les enfants sur l’utilisation et les coûts des soins de santé.

**Méthodes:**

Essai randomisé en grappes d’une voie clinique, échelonné, mené dans cinq services d’urgence en Alberta, au Canada, du 1 février au 30 novembre 2019. Le cheminement clinique mettait l’accent sur l’évaluation normalisée du risque de symptômes persistants, la fourniture de renseignements uniformes aux patients et aux familles, et l’aiguillage vers un suivi externe. Les données administratives dépersonnalisées ont permis de mesurer six résultats : visites de retour à l’urgence; visites de suivi en clinique externe; durée du séjour à l’urgence, y compris le temps total. le temps entre le triage et l’évaluation initiale du médecin, et le temps entre l’évaluation initiale du médecin et la décision; et le nombre total de demandes de remboursement du médecin dans un épisode de soins.

**Résultats:**

Un total de 2878 patients uniques (1164 femmes, 1713 hommes) âgés de 5 à 17 ans (médiane 11,00, IQR 8, 14) répondaient aux critères de cas. Ils ont effectué 3009 visites aux 5 sites et 781 visites de suivi aux soins ambulatoires, ce qui représente 2910 épisodes de soins. La mise en œuvre n’a pas modifié la probabilité d’une visite de retour à l’urgence (RC 0,77, IC à 95 %, 0,39, 1,52), mais a augmenté la probabilité de visites de suivi en clinique externe (RC 1,84, IC à 95 %, 1,19, 2,85). La durée totale du séjour à l’urgence est demeurée inchangée, mais le temps écoulé entre l’évaluation initiale du médecin et la décision a diminué considérablement (changement moyen : -23,76 minutes, IC à 95 %, -37,99, -9,52). Le nombre total de demandes de règlement de médecins a augmenté de façon significative à seulement 1 site sur 5.

**Conclusions:**

La mise en œuvre d’un cheminement clinique à l’urgence a augmenté le suivi des patients externes et réduit le temps entre l’évaluation initiale du médecin et son élimination, sans augmenter les coûts des médecins. La mise en œuvre d’un cheminement clinique peut harmoniser davantage les soins de courte durée en cas de commotion cérébrale pédiatrique avec les lignes directrices de pratique clinique existantes tout en rendant les soins plus efficaces.

## Clinician’s capsule

**Table:** 

***What is known about the topic?***
Implementation of clinical practice guidelines for pediatric concussion is inconsistent, resulting in significant practice variation.
***What did this study ask?***
Does the active implementation of a clinical pathway for acute care of pediatric concussion alter health care utilization and costs?
***What did this study find?***
Implementation reduced the time from initial assessment to disposition in the ED and increased outpatient follow-up, without increasing physician costs.
***Why does this study matter to clinicians?***
Implementation of a clinical pathway for pediatric concussion can align care with practice guidelines and increase efficiency in the ED.

## Introduction

Millions of children sustain a concussion annually in North America, and more seek medical care each year [1, 2]. Approximately 15–25% of children with concussion report persistent post-concussive symptoms, functional disability, and poorer quality of life [3–5]. Clinical practice guidelines for acute care of concussion recommend assessment of risk factors for persistent post-concussive symptoms, education about concussion self-management and return to school and sport, and referral for follow-up [6–8]. However, guideline implementation is inconsistent, and significant practice variation persists [9, 10]. This failure of knowledge translation likely occurs because practice guidelines are seldom translated into clinical pathways [11, 12] and because pathway implementation typically relies on passive dissemination rather than active, planned interventions [13, 14].

In 2015, the Maternal Newborn Child & Youth Strategic Clinical Network of Alberta Health Services (AHS), the provincial health care system, convened a workgroup to develop a clinical pathway for acute care of concussion in the emergency department (ED). We sought to test the effects of implementing the pathway on patient outcomes and health care utilization and costs using an active, planned intervention. Here, we report on the impact on health care utilization and costs. We hypothesized implementation would result in decreased likelihood of ED return visits; increased likelihood of outpatient follow-up; decreased length of stay in the ED, particularly in the time from physician initial assessment to disposition; and reduced costs of episodes of care.

## Methods

### Study design and time period

The project involved a stepped wedge, cluster randomised trial in 5 EDs, registered at ClinicalTrials.gov (NCT05095012) and reported per the relevant CONSORT extension [15]. Each site was considered a cluster. After a 2-month lead-in, implementation occurred sequentially every 2 months, such that each site contributed observations at least 2-months pre- and post-implementation. An independent biostatistician used a computerized algorithm to randomize the implementation sequence, which was revealed to all sites in September 2018. The trial began on February 1, 2019, and concluded on November 30, 2019, although administrative data were collected through February 2020 to ensure capturing all relevant episodes of care. The project was conducted under a data sharing agreement with AHS, with administrative approval from AHS and the sites, and with approval by all related research ethics boards. The analysis of health care utilization and cost was conducted with waiver of consent, because it relied on de-identified administrative data.

### Study setting

The 5 sites were Alberta Children’s Hospital and South Health Campus in Calgary, which implemented the pathway concurrently, because they shared the same group of pediatric emergency medicine physicians, and Stollery Children’s Hospital, Northeast Community Health Center, and Grey Nun's Community Hospital in Edmonton. Three sites are high-volume teaching hospitals, whereas two are community facilities with lower patient volumes.

### Intervention

The clinical pathway emphasized standardized assessment of risk for persistent post-concussive symptoms using the validated 5P clinical risk score [16], provision of consistent information about concussion to patients and families, and referral for outpatient follow-up based on the risk stratification tool [17]. When a suspected concussion was triaged, nursing staff added three items to the patient’s chart: a 5P risk score sticker (Supplemental Fig. 1); standardized leaflets containing concise, evidence-based information about concussion and return to school and sport; and a flyer regarding a project-specific website that provided developmentally appropriate content about concussion for parents and patients. Physicians were encouraged to score the 5P sticker to assist with disposition and provide the leaflets and flyer to patients and families. Local specialty concussion clinics agreed to see patients at high risk of persistent symptoms immediately upon referral. The 5P sticker encouraged referral to family physicians in all other cases.

The implementation strategy drew on the Theoretical Domains Framework [18]. We assessed organizational issues (e.g., patient workflow, health provider roles) during site visits and interviewed physicians and nurses regarding barriers and facilitators to implementation [19]. Based on this input, the implementation strategy combined a physician–nurse champion team at each site, a reminder system (i.e., adding the 5P sticker, information leaflets, and website flyer to patient charts), site champion teleconferences, and physician and nurse teaching sessions at each site before implementation. In addition, the study coordinator (HG) and director (KOY) were available for consultation.

### Cohort identification and episode of care definition

Administrative data were drawn from the Alberta Ambulatory Care Reporting System, National Ambulatory Care Reporting System, and physician claims. These databases contain information on all medical services received by publicly insured residents of Alberta. The identified cohort included children ages 5–17 who made at least one visit to the 5 sites’ EDs during the trial and received diagnoses for concussion or other head injuries, consistent with the Centers for Disease Control and Prevention surveillance definition (see Supplemental Table 1 for codes and rationale) [20, 21]. Episodes of care were defined based on review of administrative data for all concussion-related visits in Alberta over a 10-year period and in consultation with study physicians [22]. An episode of care includes an index ED visit and all subsequent visits to ED or outpatient settings within designated timeframes. An AHS data analyst (OB) completed cohort identification and data management prior to independent statistical analysis (KT).

### Outcome measures

Administrative data were used to assess 6 outcomes: ED return visits; outpatient follow-up visits; total length of stay; time from triage to physician initial assessment; time from initial physician assessment to disposition; and total physician claims per episode of care (see Supplemental Table 2 for definitions).

### Statistical power

To determine statistical power, we estimated the detectable proportion difference and Cohen’s *d* for a two-tailed comparison of independent groups, as an approximation to our analyses. The data included 2910 episodes of care containing an ED visit. For binary outcomes, assuming a proportion of 0.032 pre-implementation (based on our data, where 32/993 episodes of care had a return ED visit pre-implementation), we had 0.80 power to detect a proportion of < 0.016 or > 0.054 post-implementation. For continuous outcomes, we had 0.80 power to detect a Cohen’s *d* of 0.11.

### Data analysis

For each outcome, we applied two statistical modeling approaches. To test the overall implementation effect, we fitted a multivariable generalized linear mixed model (GLMM) for continuous outcomes or generalized linear model (GLM) for categorical outcomes, with a binary intervention term (‘pre’ vs. ‘post’ implementation) as the main predictor. To examine outcomes relative to implementation timing, we adopted an interrupted time-series approach using a ‘time’ indicator to represent the number of months before or after implementation, with segments spanning 3–4 months, and then fitted generalized least squares (GLS) for continuous outcomes or a GLM for binary or ordinal outcomes, using linear splines [23, 24]. We focused on the specific contrast of the segments immediately preceding and following implementation. For GLMM or GLS, a within-subject correlational structure was specified for the intercept, with patient and episode of care as grouping factors. For logistic or ordinal GLM, robust standard errors were estimated with patient as the clustering unit.

Models included the following covariates of methodological or substantive importance while maintaining an approximate 10:1 observation-to-parameter ratio: site; diagnosis; triage level; distance to site; neighborhood socioeconomic status [25]; child’s age at injury and sex; and calendar time (see Supplemental Table 3 for coding). In overall models, we initially tested two interactions, of implementation with site and diagnosis, that were dropped if *p* > 0.05. For continuous predictors, we included quadratic terms in GLMMs or applied restricted cubic splines for GLS or GLMs [26]. For each model, listwise deletion was performed for missing values on the outcome or any covariates; 9.0 to 13.6% of observations were excluded for missingness. All analyses were performed using *R* version 4.0.3, using functions from the *rms* package [27, 28]. Detailed statistical results are available from the corresponding author.

## Results

Table 1 provides descriptive information. Across the 10-month trial, 2878 unique patients (1164 female, 1713 male, 1 sex unknown) with a median age of 11 years (IQR 8, 14) met case criteria. They completed 3009 visits to the 5 sites (1023 before and 1986 after implementation) and 781 follow-up visits to outpatient settings. The visits constituted 2910 episodes of care; only 30 patients had more than one episode. Most episodes (2289, 78.7%) involved a single visit. Concussion or post-concussion syndrome was diagnosed at 1847 (61.4%) of all ED visits. Table 2 summarizes unadjusted outcome data.

**Table 1:** Descriptive information including covariates, prior to and after pathway implementation, at visit, episode of care, and unique patient levels

ED visit level	*N*	Pre-implementation (*n* = 1026)	Post-implementation (*n* = 1983)
Site, frequency (%)	3009		
Alberta Children's Hospital		291 (23.1)	1,152 (43.2)
South Health Campus		71 (5.6)	304 (11.4)
Grey Nuns Community Hospital		55 (4.4)	83 (3.1)
Northeast Community Health Centre		106 (8.4)	34 (1.3)
Stollery Children's Hospital		503 (39.9)	410 (15.4)
Triage disposition, frequency (%)	3007		
Non-urgent/semi-urgent		333 (32.5)	728 (36.8)
Urgent		514 (50.1)	885 (44.7)
Emergent/resuscitation		179 (17.5)	368 (18.6)
Travel distance (km), median (IQR)	2992	13.00 (8.00, 21.00)	14.00 (9.00, 21.00)
Diagnosis, frequency (%)	3009		
Concussion		613 (59.7)	1,183 (59.7)
Post-concussion syndrome		24 (2.3)	27 (1.4)
Other specified injuries of head		28 (2.7)	37 (1.9)
Unspecified injury of head		361 (35.2)	736 (37.1)
Nature of injury, frequency (%)	3009		
Isolated head injury		836 (81.5)	1,566 (79.0)
Polytrauma		190 (18.5)	417 (21.0)
Number of imaging requisitions, frequency (%)	3009		
0		870 (84.8)	1,731 (87.3)
1		112 (10.9)	166 (8.4)
2		28 (2.7)	52 (2.6)
3 or more		16 (1.6)	34 (1.8)
Disposition, frequency (%)	3009		
Admitted to hospital		17 (1.7)	27 (1.4)
Discharged home		952 (92.8)	1,885 (95.1)
Other (e.g., transferred, left against medical advice)		57 (5.6)	71 (3.6)

Episode of care level	*N*	Pre-implementation (*n* = 993)	Post-implementation (*n* = 1,917)
Total number of visits within episode of care, mean (SD)	2910	1.27 (0.73)	1.39 (1.06)
Total number of visits following 1st ED visit, mean (SD)	2910	0.23 (0.67)	0.34 (1.02)

Unique patient level	*N*	Pre-implementation (*n* = 976)	Post-implementation (*n* = 1885)
Age (lowest recorded), median (IQR)	2861	11.00 (8.00, 14.00)	11.00 (8.00, 15.00)
Sex, frequency (%)	2861		
Female		393 (40.3)	763 (40.5)
Male		583 (59.7)	1,121 (59.5)
Other or Unknown		0 (0.0)	1 (0.1)
Material deprivation index [25] quantile, median quantile (IQR)	2612	3.00 (2.00, 4.00)	2.00 (2.00, 4.00)
Total number of EOCs for the patient, frequency (%)	2861		
1		969 (99.3)	1,879 (99.7)
2		7 (0.7)	6 (0.3)
Total number of visits (across all EOCs) for the patient, median (IQR)	2861	1.00 (1.00, 1.00)	1.00 (1.00, 1.00)

*IQR* Interquartile range

Patient level data excludes 17 children who had two or more episodes of care, which occurred both before and after implementation

**Table 2:** Outcomes prior to and after pathway implementation at visit and episode of care levels, unadjusted for covariates

ED visit level	*N*	Pre-implementation (*n* = 1026)	Post-implementation (*n* = 1983)
Total length of stay (minutes), median (IQR)	3008	170.50 (117.00, 243.00)	152.00 (102.00, 220.00)
Time from triage to physician initial assessment (minutes), median (IQR)	2866	94.80 (49.20, 154.20)	82.20 (43.80, 135.00)
Time from physician initial assessment to disposition (minutes), median (IQR)	2865	49.20 (25.20, 111.30)	46.80 (24.00, 93.00)

Episode of care level	*N*	Pre-implementation (*n* = 993)	Post-implementation (*n* = 1917)
Number of ED return visits following 1st ED visit, frequency (%)	2910		
0		961 (96.8)	1855 (96.8)
1		31 (3.1)	58 (3.0)
2		1 (0.0)	4 (0.0)
Number of outpatient follow-up visits following 1st ED visit, frequency (%)	2910		
0		871 (87.7)	1583 (82.6)
1		79 (8.0)	243 (12.7)
2 or more		43 (4.3)	111 (5.8)
Physician claims for the episode of care (CAD), mean (SD)	2910	207.20 (146.76)	228.06 (157.25)

*IQR* interquartile range

### Return visits

**Fig. 1:**
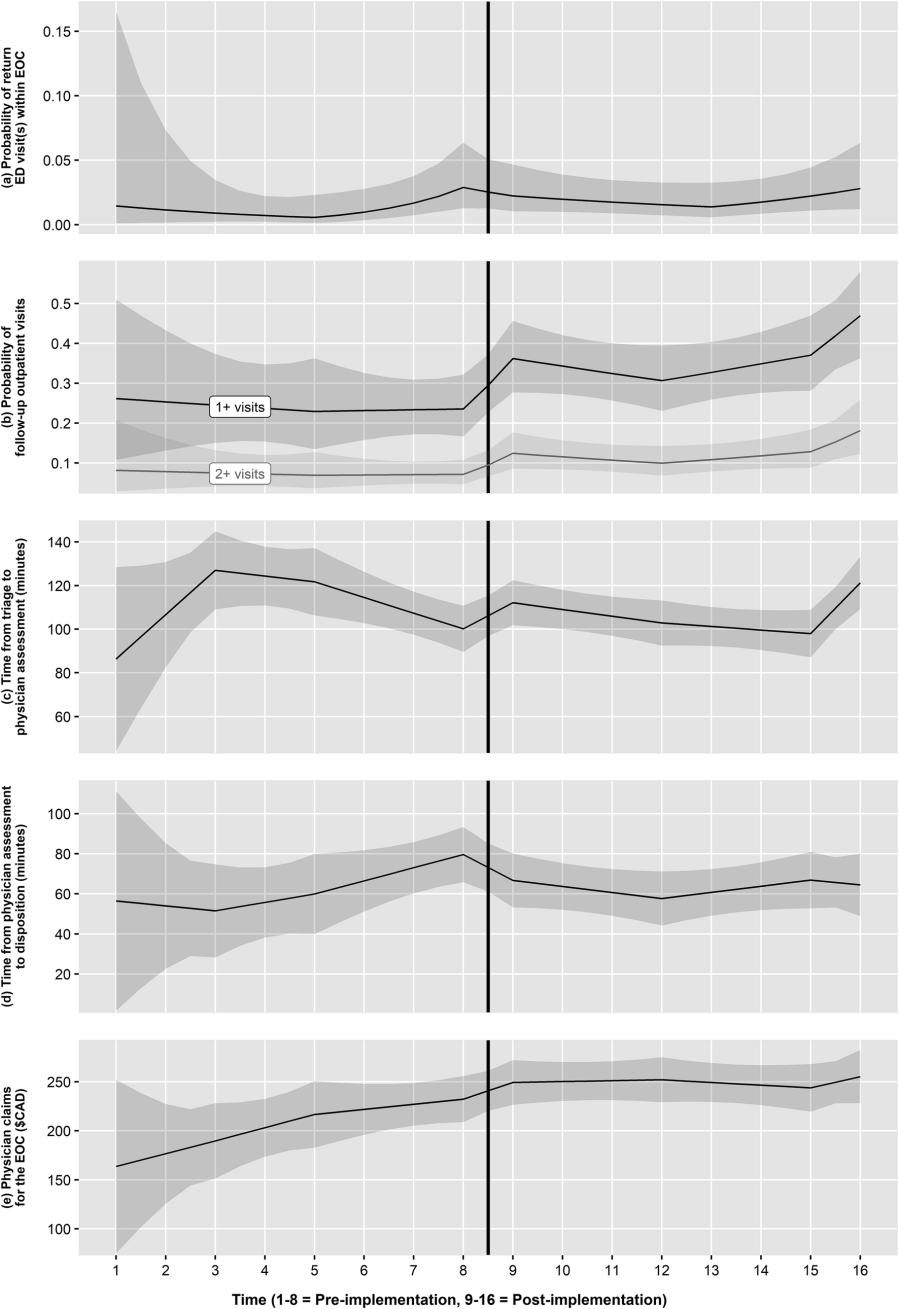
**a**–**e** Segmented model estimates of health care utilization and cost outcomes, adjusted for covariates, across time segments pre- and post-implementation, with 95% confidence intervals (shaded). **a** Probability of return ED visit(s) within an episode of care (EOC). **b** Probability of follow-up outpatient visit within an episode of care; **c** Time from triage to physician initial assessment per visit (minutes); **d** Time from physician initial assessment to disposition per visit (minutes); **e** Total physician claims for an episode of care

Only 94 of 2910 episodes of care included ED return visits. Implementation did not affect return visits in the overall model or across the pre- to post-implementation contrast in the segmented model (Fig. 1a). Return visits were more likely for diagnoses of concussion than other head injuries (OR 2.13, 95% CI 1.29, 3.53).

### Follow-up visits

The overall effect of implementation on the number of outpatient follow-up visits was not significant, although the odds of one or more follow-up visits were 1.42 times higher after implementation (95% CI 0.97, 2.09). The pre- to post-implementation contrast was significant in the segmented model, with 1.84 times higher odds of one or more follow-up visits (95% CI 1.19, 2.85; Fig. 1b). Follow-up visits were more common after diagnoses of concussion than other head injuries, in older than younger children, and in patients residing in neighborhoods of higher socioeconomic status and at longer distances.

### Length of Stay

The overall effect of implementation on total length of stay was not significant (mean change − 10.25 min, 95% CI − 26.68, 6.18), nor was the specific contrast of pre- versus post-implementation in the segmented model (mean change 0.53 min, 95% CI − 14.47, 15.54). Time from triage to physician initial assessment increased significantly after implementation in the overall model at 3 of 5 sites (intervention X site interaction, Wald *χ*^2^ = 10.07, *p* = 0.039), as well as in the pre- to post-implementation contrast in the segmented model (mean change 11.99 min, 95% CI 2.04, 21.94; see Fig. 1c). Time from physician initial assessment to disposition decreased significantly after implementation in the overall model (mean change − 23.76 min, 95% CI − 37.99, − 9.52), as well as across the pre- to post-implementation contrast in the segmented model (mean change − 12.94 min, 95% CI − 25.84, − 0.04; see Fig. 1d).

Longer lengths of stay, as well as longer times from physician initial assessment to disposition, were associated with higher acuity triage and diagnoses of concussion relative to other head injuries. Longer times from physician initial assessment to disposition were also associated with younger and older ages at injury and longer travel distances. Longer times from triage to physician initial assessment were associated with lower acuity triage and shorter travel distances. All three length of stay variables were related to calendar time.

### Physician costs

Total physician claims per episode of care increased significantly after implementation in the overall model at only one site (intervention X site interaction, Wald *χ*^2^ = 10.22, *p* = 0.037). The specific pre- to post-implementation contrast was not significant in the segmented model (mean change $17.02, 95% CI − 5.00, 39.03; see Fig. 1e). Higher total claims were associated with diagnoses of concussion relative to other head injuries and longer travel distances.

## Discussion

### Interpretation

In this trial, implementation of a clinical pathway for acute care of pediatric concussion increased outpatient follow-up and reduced the time from physician initial assessment to disposition, although not overall length of stay. Thus, pathway implementation appears to have aligned care more closely with existing practice guidelines, which emphasize follow-up care as key to improving pediatric concussion outcomes [6–8], and also made care more efficient.

### Previous studies

No other published randomized trial has tested the impact of implementing a clinical pathway for pediatric concussion in the ED, although a prior quality improvement study showed a simple intervention increased the completeness of discharge instructions [29]. In our study, the design and implementation of the clinical pathway were guided by an evidence-based, theory-driven approach, but also relied on extensive input from site physicians and nurses. Clinical pathway implementation is more effective when both the pathway and its implementation are co-designed with health care providers [13, 30]. The implementation strategy was intended to work within existing hospital resources, minimize reliance on study support, ensure feasibility and sustainability, and permit evaluation of implementation in a realistic context. We believe these goals were achieved given the consistent effects of implementation across 5 sites that varied substantially in staffing patterns, patient volumes, research support, and other factors that can affect clinical pathway uptake [31].

Although the effects of pathway implementation did not vary by diagnosis, concussion diagnoses were associated with longer times from physician initial assessment to disposition, an increased likelihood of return ED visits and follow-up outpatient visits, and higher physician claims, compared to other head injury diagnoses. Thus, a diagnosis of concussion was associated with more intensive and costly health care utilization. However, unspecified injury of head is often diagnosed in the ED rather than concussion despite documented evidence of traumatic brain injury, and, therefore, was historically included in CDC surveillance definitions of traumatic brain injury [32]. Research is needed on factors that determine the diagnostic coding of head trauma, as are clearer guidelines for diagnosis of concussion, especially in younger children [33].

### Strengths and limitations

Study strengths include the stepped wedge, cluster randomized study design, diverse ED settings, large sample, and comprehensive statistical modeling. Limitations include that the clinical pathway was designed for ED settings and might not yield similar changes in outpatient settings. Our episode of care definition was based on epidemiological data and expert opinion [20], but different criteria could alter the findings. Administrative data is of uncertain quality and inaccurate coding is potentially unavoidable [34]. Cost data were restricted to physician claims and excluded the direct costs of care by other providers and any indirect costs. More children were seen after than before the intervention, so parameter estimates in our models were more precise post-implementation than pre-implementation. The inclusion of other head injury diagnoses in the case definition may have resulted in the cohort including some children without concussion, although intervention effects did not vary by diagnosis (see Supplemental Table 2). We had limited information about the nature of follow-up care (e.g., concordance with 5P sticker score; see Supplemental Tables 4 and 5).

### Clinical implications

Our findings suggest that implementing a relatively simple clinical pathway in the ED can promote outpatient follow-up, in accordance with existing practice guidelines [6–8], while enabling physicians to care for children with concussion more efficiently. By standardizing the guidance offered to patients and families, the clinical pathway also likely helps to reduce practice variation in the acute care of pediatric concussion [9, 10].

### Research implications

A key next step will be to examine the effect of pathway implementation on patient outcomes. We originally intended to examine patient outcomes but did not obtain enough data on the study website to do so. Further qualitative study of both implementation and outcomes also is indicated to better understand the effects of the intervention [19]. Another important step will be examining the effectiveness of clinical pathways designed for primary care physicians, who provide an increasing proportion of care for children with concussion [35, 36].

## Conclusion

Meaningful changes in concussion care—i.e., decreased time from physician initial assessment to disposition and increased outpatient follow-up—resulted from the planned implementation of a clinical pathway in the ED, without increased physician costs. These changes in care are likely an important step to better patient outcomes.

## Supplementary Information

Below is the link to the electronic supplementary material.

Supplementary file1 (DOCX 296 KB)

## Data Availability

The de-identified administrative data used for this analysis will not be made available based on the data agreement with Alberta Health Services.

## Abbreviations

AHS: Alberta Health Services
ED: Emergency department
GLM: Generalized linear model
GLMM: Generalized linear mixed model
GLS: Generalized least squares
