# Supplementary material for: Health care impact of implementing a clinical pathway for acute care of pediatric concussion: a stepped wedge, cluster randomised trial
Source: CJEM. 2023 Jun 23;25(7):627–36. doi: 10.1007/s43678-023-00530-1 (PMC10333406; doi:10.1007/s43678-023-00530-1)
Supplement: Supplementary file 1 — Supplementary file1 (DOCX 296 KB) [file 43678_2023_530_MOESM1_ESM.docx]

**Online Supplementary Material**

Supplemental Table 1: Diagnostic codes used for case identification

Rationale for Diagnostic Code Selection

Supplemental Table 2: Administrative data outcome measures and definitions

Supplemental Table 3: Covariates in statistical models

Supplemental Table 4: Comparison of follow-up visit location pre-implementation versus post-implementation

Supplemental Table 5: Comparison of days to initial follow-up visit within an episode of care pre-implementation versus post-implementation

Follow-Up Care Analyses

Supplemental Figure 1: 5P risk score sticker

Supplemental Table 1: Diagnostic codes used for case identification

| **Location** | **Source** | **Codes** |
| --- | --- | --- |
| Emergency Departments | ICD-10 | F07.2 (“postconcussional syndrome”) |
|  |  | S09.8 (“other specified injuries of head”) |
|  |  | S09.9 (“unspecified injury of head”) |
|  |  | Any of 13 diagnostic codes beginning with S06.0 (“concussion”) |
| All Outpatient Settings | ICD-9 | 850 (“concussion”) |
|  |  | 310.2 (“postconcussional syndrome”) |

ICD = International Statistical Classification of Diseases and Related Health Problems.

Note. ICD-10 was used for visits to Emergency Departments and ICD-9 was used for visits to outpatient settings.

Rationale for Diagnostic Code Selection

Up until 2017, the Centers for Disease Control and Prevention (CDC) included the diagnosis of “unspecified injury of head” (ICD-9 959.01; ICD-10 S09.9) in their surveillance definition of concussion/traumatic brain injury.^1,2^ We expected that children who received the S09.9 diagnosis would be triaged based on concerns about their head injury and managed according to the clinical pathway; our informal discussions with ED physicians on our research team suggested the diagnosis is often used when a concussion was suspected but could not be definitively confirmed, especially in younger children. Thus, we expected that children with the “unspecified injury of head” diagnosis would show changes following pathway implementation like those shown by patients with a formal concussion diagnosis. We tested that assumption by including diagnosis X intervention interaction terms in our overall models, and none of them were significant. After we submitted the grant application for the parent project in 2016, the CDC proposed a new surveillance definition for ICD10 that excluded the unspecified injury of head code.^3^ However, in a subsequent study supported by the CDC, a multisite medical record review of ED visits for unspecified injury of head following the ICD10 coding transition found that about 50% of those records had medium or high evidence of traumatic brain injury.^4^ The authors concluded, “Exclusion of the S09.90 code in surveillance estimates may result in many missed TBI cases; inclusion may result in counting many false positives.” To address this concern, we repeated all our primary analyses but limited the sample to children with a concussion diagnosis. The findings were largely unchanged, although some effects were more pronounced or consistent when restricted to the concussion group (i.e., the increase in time from triage to physician initial assessment was significant across sites; the increase in follow-up visits was significant in the overall model; the increase in physician claims was significant across sites). Given that the study protocol called for the inclusion of children with the S09.9 diagnosis, and that none of the diagnosis X intervention interaction terms were significant, we chose to report the full analysis in the main paper.

References

1. National Center for Injury Prevention and Control. (2003). *Report to Congress on mild traumatic brain injury in the United States: Steps to prevent a serious public health problem*. Atlanta, GA: Centers for Disease Control and Prevention.

2. Faul M, Xu L, Wald MM, Coronado VG. *Traumatic brain injury in the United States: emergency department visits, hospitalizations and deaths 2002–2006*. (2010). Atlanta, GA: Centers for Disease Control and Prevention, National Center for Injury Prevention and Control.

3. Hedegaard HB, Johnson RL, Ballesteros MF. (2017). Proposed ICD–10–CM surveillance case definitions for injury hospitalizations and emergency department visits. *National health statistics reports, 100.*

4. Peterson A, Babella BA, Johnson J, Hume B, Liu A, Costich JF et al. (2021). Multisite medical record review of emergency department visits for unspecified injury of head following the ICD-10-CM coding transition. *Inj Prev, 27*, i13-i18.

Supplemental Table 2: Administrative data outcome measures and definitions

| **Variable** | **Definition** |
| --- | --- |
| Emergency department return visits | Number of additional visits to the emergency department after an initial visit within an episode of care |
| Outpatient follow-up visits | Number of outpatient visits following an emergency department visit to a physician office or ambulatory clinic within an episode of care |
| Length of stay | Total time spent during emergency department visit (minutes) |
|  | Time from initial triage to physician initial assessment (minutes) |
|  | Time from physician initial assessment to disposition (minutes) |
| Total physician claims per episode of care | Total costs of all physician claims for a given episode of care (CAD) |

Supplemental Table 3: Covariates in statistical models

| **Covariate** | **Coding** |
| --- | --- |
| Site | Dummy variables referenced to Alberta Children’s Hospital |
| Diagnosis | Concussion/post-concussion syndrome versus other head injury |
| Triage level | Non-urgent/semi-urgent, urgent, and emergent/resuscitation |
| Distance to site | Natural log of distance from home address to site (km) |
| Neighborhood socioeconomic status | Pampalon material deprivation index (quintile) ^25^ |
| Child’s age at injury | Years |
| Child’s sex | Male versus female |
| Calendar time* | Month from start to end of trial |

*Omitted from segmented models due to circularity with site and time segment covariates

Supplemental Table 4: Comparison of follow-up visit location pre-implementation versus post-implementation

| **Visit location, freq (%)** | **Pre-implementation (n=193)** | **Post-implementation (n=588)** |
| --- | --- | --- |
| Ambulatory clinic | 8 (4.1) | 53 (9.0) |
| Physician office | 185 (95.9) | 625 (91.0) |

Supplemental Table 5: Comparison of days to initial follow-up visit within an episode of care pre-implementation versus post-implementation

| **Days** **to** **initial** **follow-up** **visit** **after** **1st** **ED** **visit** **within** **episode of care** | **Pre-implementation** **(n=122)** | **Post-implementation** **(n=354)** |
| --- | --- | --- |
| Median (IQR) | 7.00 (3.25, 11.00) | 7.00 (4.00, 11.00) |
| Mean (SD) | 8.81 (7.30) | 8.17 (6.54) |

Follow-Up Care Analyses

Reviewers asked for additional information regarding the nature of the follow-up care received by patients in the trial. Specifically, reviewers asked about the correspondence of patient’s 5P scores and follow-up care (i.e., did high risk patients attend specialty clinics), and were patients seen for follow-up within recommended intervals. Because we had limited information about follow-up and these outcomes were not a focus of the study protocol, we have presented them in the online appendix.

We did not have patient’s 5P scores available to us in the administrative data, so we could not address the correspondence of follow-up location to the 5P score. Additionally, the coding of follow-up location did not differentiate speciality clinics from other ambulatory clinics, but only differentiated ambulatory clinics from physician offices. We compared the percentage of follow-up visits to ambulatory clinics versus physician offices pre- versus post-implementation (see Supplemental Table 4); we found a significant unadjusted increase in the percentage that involved ambulatory clinics (*p* = .029). This result may suggest more speciality referrals, but we cannot be sure that this is the case. We also examined time to the first outpatient follow-up visit within an episode of care and found it was unchanged by the intervention, with a median of 7 days both pre- and post-implementation (IQR pre-implementation 3.25-11, post-implementation 4-11). Follow-up within a week would be consistent with most clinical practice guidelines.

In summary, the intervention increased the proportion of children who received follow-up and appears to have made follow-up in ambulatory clinics relatively more common. The intervention did not change the timing of follow-up, which was within recommended intervals before and after the intervention for most children who had follow-up.

Supplemental Figure 1: 5P risk score sticker
